# Impact of COVID-19 pandemic on the surgical treatment of gastric cancer

**DOI:** 10.6061/clinics/2021/e3508

**Published:** 2021-11-17

**Authors:** Amanda Juliani Arneiro, Marcus Fernando Kodama Pertille Ramos, Marina Alessandra Pereira, André Roncon Dias, Bruno Zilberstein, Ulysses Ribeiro, Sergio Carlos Nahas

**Affiliations:** Departamento de Gastroenterologia, Hospital das Clinicas HCFMUSP, Faculdade de Medicina, Universidade de Sao Paulo, Sao Paulo, SP, BR.

**Keywords:** Stomach Neoplasms, Coronavirus, Coronavirus Disease of 2019, SARS-CoV-2, Surgical Treatment, Postoperative Complications

## Abstract

**OBJECTIVE:**

The Coronavirus Disease 2019 (COVID-19) pandemic has been recognized as one of the most serious public health crises. This study aimed to evaluate the short-term impact of the pandemic on the surgical treatment of patients with gastric cancer (GC) in addition to their clinicopathological characteristics. We also verified adherence to the COVID-19 screening protocol adopted in the institution.

**METHODS:**

All patients with GC who underwent surgical treatment between 2015 and 2021 were retrospectively evaluated and divided into two groups according to the time period: control group (2015-2019) and COVID group (2020-2021). The institutional protocol recommends that patients referred for surgery undergo RT-PCR for severe acute respiratory syndrome coronavirus 2 infection.

**RESULTS:**

A total of 83 patients were classified into the COVID group and 535 into the control group. The number of surgical procedures performed in the control group was 107 (SD±23.8) per year. Diagnostic procedures (*p*=0.005), preoperative chemotherapy (*p*<0.001), and adenocarcinomas without Lauren's subtype (*p*=0.009) were more frequent in the COVID group than in the control group. No significant difference was observed in the pathological characteristics and surgical outcomes of curative GC between the two groups. Evaluation of protocol compliance showed that of 83 patients with GC in the COVID group, 19 (22.9%) were not tested for COVID-19 before surgery. Two patients tested positive for COVID-19 (one preoperative and one postoperative).

**CONCLUSION:**

A decrease in the average number of surgeries and a higher frequency of diagnostic procedures occurred during the pandemic than in the previous time period. Tumor/node/metastasis classification, morbidity rates, and mortality rates in patients with GC during the pandemic did not differ from those in the previous time period. Accordingly, GC surgical treatment with acceptable screening protocol compliance could be safely performed during the COVID-19 pandemic.

## INTRODUCTION

Severe acute respiratory syndrome coronavirus 2 (SARS-CoV-2) belongs to a large family of coronaviruses and is responsible for the disease known as coronavirus disease 2019 (COVID-19), first detected in Wuhan, China, in December 2019. In 2020, the World Health Organization (WHO) declared COVID-19 an international public health emergency (1), and in March 2020, WHO declared a pandemic. Since then, COVID-19 has remained a significant cause of death. More than 4 million people have died from the disease worldwide. The first confirmed case in Brazil was in February 2020, and the first COVID-19-related death was recorded in March 2020 in São Paulo (2,3).

The spread of COVID-19 has affected the socioeconomic and health systems of many countries, including Brazil. The pandemic caused a health system crisis leading to an increase in the occupation of hospital beds. This situation resulted in maximum occupancy rates in intensive care units (ICUs), consequently restricting access of patients with other diseases to the health system. Other important causes of morbimortality, such as cancer, remained active during this period. Thus, restricted access to the health system may have impaired diagnosis of new cases, thereby reducing the chances of detecting the disease at less advanced stages and delaying treatment initiation (3-5).

Among tumors with a high mortality rate, gastric cancer (GC) remains the fourth leading cause of cancer-related death worldwide (6). In 2019, 15,111 deaths from stomach cancer occurred in Brazil, representing a mortality rate of 7.39 per 100,000 inhabitants. The Brazilian National Cancer Institute estimates an incidence of 21,230 new cases of GC among men and women for each year in the triennium of 2020-2022 (7).

A retrospective study recently conducted in China revealed that admissions of patients with GC during the COVID-19 pandemic decreased by 30%, accompanied by an increase in waiting time for surgery. In addition, longer hospital stays were reported suggesting a change in the standard treatment for GC during this period (8). Patients with cancer are already considered more vulnerable to other diseases mainly because of immunosuppression, low performance status, and nutritional deficiency. Accordingly, it has been suggested that COVID-19 patients with cancer exhibit more severe symptoms and have worse outcomes than those without cancer (9). Additionally, patients with cancer had an increased risk of developing severe infection and subsequent complications, especially if surgery or chemotherapy was performed 1 month before COVID-19. During this time period, diagnoses of COVID-19 patients with cancer were associated with higher ICU admissions and risk of death than of those without cancer (10,11). Moreover, a study with more than 20,000 patients demonstrated an increased risk of COVID-19 among oncological patients, with a higher risk attributed to elderly male patients and those over 65 years of age (12).

At the Cancer Institute of the State of São Paulo, an oncology reference center (13), measures were adopted at the beginning of the pandemic to prevent COVID-19 from hospital admission to discharge. An institutional screening protocol was established for the hospitalization of surgical patients consisting of an RT-PCR-based SARS-CoV-2 infection test by nasopharynx swab, remote outpatient medical appointments (telemedicine), and restriction in the number of visits to hospitalized patients. These measures were introduced to reduce the circulation of individuals in the hospital. Certain measures related to surgical procedures, such as slow deflation of pneumoperitoneum and removal of trocars were also performed during laparoscopic surgeries to reduce the dispersion of aerosols. Finally, similar to other centers, elective surgeries for non-cancer indications such as intestinal reconstructions and hernia repairs were postponed during the pandemic (14,15).

The impact on cancer treatment results because of restrictions and surgery postponements in patients with GC remains unknown. Thus, the present study aimed to evaluate the short-term impact of the COVID-19 pandemic on the surgical treatment and clinicopathological characteristics of patients with GC. Additionally, as a secondary objective, we examined adherence to the preoperative screening protocol for COVID-19 adopted at the institution during the pandemic period.

## METHODS

Patients with gastric adenocarcinoma who underwent surgical treatment from 2015 to 2021 were retrospectively evaluated. Surgical procedures included curative, palliative, and diagnostic procedures. Patients who underwent gastric surgeries as part of a procedure for other primary tumors, non-adenocarcinoma tumors, and those not related to GC treatment were excluded.

Patients were divided into two groups according to the date of the procedure: control group, between 2015 and 2019; and COVID group, between March 2020 and March 2021, (the period that included the first year of the COVID-19 pandemic in Brazil).

Clinical data included age, sex, body mass index, presence of comorbidities according to the Charlson-Deyo comorbidity index (CCI) (16) (without the inclusion of age and cancer in the score), American Society of Anesthesiologists (ASA) classification, and preoperative chemotherapy (CMT), including neoadjuvant and perioperative regimens.

The evaluated outcomes included surgical treatment (palliative *versus* curative intent *versus* diagnostic), surgical access (laparoscopic *versus* open), number of resected lymph nodes, average number of surgeries, and a higher frequency of diagnostic procedures. The tumor/node/metastasis (TNM) stage, postoperative complications (POCs), mortality rate, and length of hospital stay were recorded. POC was graded according to the Clavien-Dindo classification, and Clavien ≥3 complications were considered a major complication (17).

Tumors were staged according to the TNM 8^th^ edition and simplified into stages I, II, III, and IV (18). The surgical technique, type of resection, and dissected lymph node chains followed the recommendations of the Japanese Gastric Cancer Association (JGCA) (19) and the Brazilian Consensus on Gastric Cancer (20,21).

The adopted institutional protocol specified that patients with GC referred for surgery should undergo the RT-PCR SARS-CoV-2 infection test by nasopharynx swab up to 7 days before the procedure. Computed tomography (CT) of the chest was mandatory at the beginning of the protocol, but throughout the year, the protocol was modified and it was performed only in selected cases. Patients with a negative test were considered eligible for surgery. In addition, respiratory symptoms related to COVID-19 during the postoperative hospitalization and follow-up appointments were monitored, and patients suspected of infection were tested again.

Statistical analyses were performed using the statistical program SPSS (version 20.0; SPSS Inc., Chicago, IL, USA), and results are presented as absolute numbers (mean, median, standard deviation, and interquartile interval) and percentages. The chi-square test was applied to verify the association between qualitative variables, and the Student’s t-test or Mann-Whitney test was performed to evaluate quantitative variables. Results were considered statistically significant at *p*<0.05.

This study was approved by ethical committee and registered under the CAAE number 44352421.2.0000.0068.

## RESULTS

### Study patients

**Figure 1:**
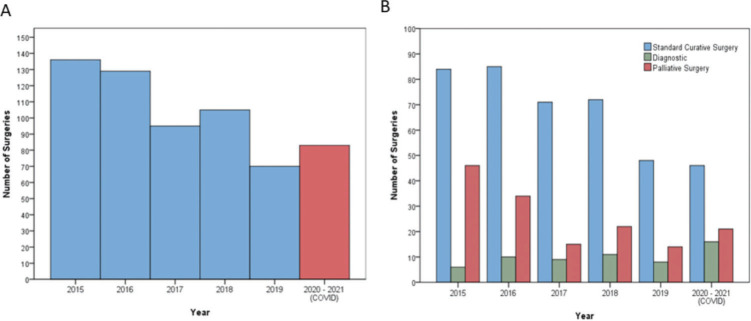
**(A)** Total surgical procedures performed between 2005 and 2021. **(B)** Intention of treatment: curative surgery, palliative surgery, or diagnostic procedures.

From 2015 to 2021, a total of 618 patients with GC underwent surgical procedures. Of these, 83 were treated during the year of the COVID-19 pandemic and were classified into the COVID group. The remaining 535 patients constituted the control group (*i.e.*, underwent surgery in the previous 5 years). The average number of surgical procedures per year in the control group was 107 (SD±23.8). Figure 1 demonstrates the distribution of procedures and types of indications during the evaluated period.

Table 1 presents the clinicopathological and surgical characteristics of both groups. We found no significant difference between the groups in sex, age, presence of comorbidities, and TNM stage. The frequency of diagnostic procedures (*p*=0.005), number of patients without specification of Lauren's histological type (*p*=0.009), and number of referrals to preoperative chemotherapy (*p*<0.001) were higher in the COVID group than those in the control group.

### Curative intent surgery

No significant differences in clinical characteristics were found between the groups among patients who underwent gastrectomy with curative intent (Table 2). The pT, pN, and final pTNM stages were similar between both time periods. The number of resected lymph nodes was higher in the COVID group than in the control group (47.4% *versus* 40.8%; *p*=0.026).

Evaluation of surgical characteristics and postoperative outcomes revealed no significant differences between the COVID and control groups in surgical access, type of gastrectomy, lymphadenectomy, length of hospital stay, surgical complications, or mortality (Table 3).

Table 4 demonstrates the POCs of the patients in the COVID group who underwent curative gastrectomy. Only 10 patients had major POCs. None of the POC cases was related to COVID-19.

### Screening protocol for COVID-19

**Figure 2:**
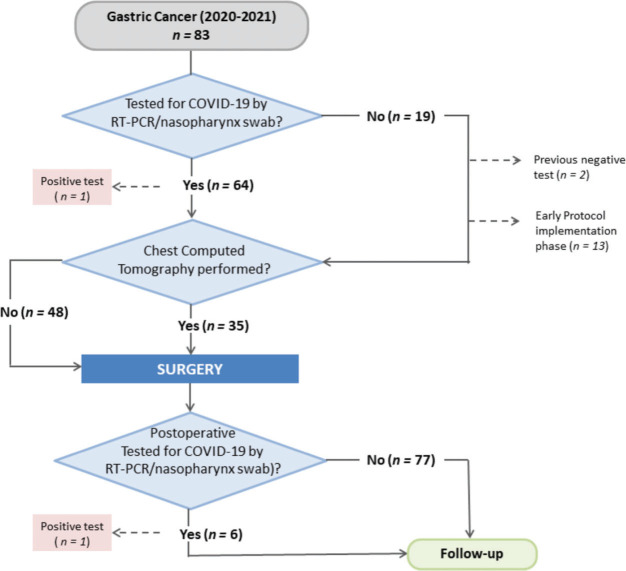
Flowchart illustrating the COVID-19 screening protocol employed at the institution.

The tests and follow-up procedures performed in the COVID group according to the screening protocol are summarized in Table 5. Among the 83 patients in the COVID group, 64 were tested by RT-PCR, which represented an adherence rate to the established protocol of 77.1%. Of these, 34 (54%) also underwent chest CT as a screening examination in the preoperative setting. One patient underwent chest CT without RT-PCR. Nineteen patients were not tested for COVID-19 preoperatively. Of these, 13 patients underwent surgery at the beginning of the pandemic when the protocol was in the implementation phase. Two patients had already tested positive for COVID-19 4 months before surgery and did not undergo a new test. The remaining four patients underwent surgery during the current period of the pandemic, without any screening examination (Figure 2).

One patient tested positive for COVID-19 preoperatively, and surgery was subsequently rescheduled. During the postoperative hospitalization, six patients were tested again and one patient tested positive for COVID-19 but was asymptomatic without POCs. During follow-up after hospital discharge, 12 patients were tested and 3 were found positive for COVID-19.

## DISCUSSION

In the present study, we evaluated surgical treatment and outcomes of patients with GC during the first year of the pandemic and compared the results with those of the previous 5 years as the control group. Our results showed a decrease in the total number of surgical procedures in 2020-2021, with an increase in the number of diagnostic surgeries. Consequently, fewer curative surgeries were performed in the COVID group. Moreover, we observed an increased number of patients who were referred for preoperative CMT. However, there were no differences in TNM staging and occurrence of POCs between the two time periods. The institutional screening protocol during the pandemic had an adhesion rate of 77.1%, with one patient positive for COVID-19 identified preoperatively and one postoperatively. However, no differences were found between the two evaluated time periods in other surgical outcomes, demonstrating that GC surgical treatments performed during the COVID-19 pandemic resulted in similar morbidity and mortality rates.

Since the restrictions were introduced, the primary issue regarding the impact of the pandemic on the health service was the decline in the number of GC cases referred for surgical treatment. Indeed, there was a decrease in the number of surgical treatments performed in the COVID group compared with that in the previous years (control group). Notably, in 2019, the mean number of surgical treatments was lower than it was in the COVID cohort because the hospital faced a financial contingency of its budget in that specific year. We found a significant increase in the indication for preoperative CMT in the pandemic period. This increase was also reflected by a higher number of diagnostic procedures, since our service includes diagnostic laparoscopy before the indication for preoperative CMT. In addition, a greater number of cases with no definition of adenocarcinoma histological subtype were identified, confirming that diagnoses were achieved mainly through biopsies and not through surgical specimen resections. It is sometimes not possible to define the exact tumor histological subtype in biopsies, mostly due to the low tumor representation associated with GC heterogeneity. In addition, a new endoscopic biopsy was indicated only in selected cases with no histological confirmation of adenocarcinoma to avoid exposing the patient to additional examinations and risk of infection.

Another concern regarding the impact of the pandemic was the possibility that patients with GC would present worse clinical performance and more advanced tumors because of the delay in seeking medical care for investigation of symptoms and in performing diagnostic exams (22). However, there were no significant differences between the groups in clinical performance. Similarly, no significant differences in TNM stages were observed in patients during the pandemic. This result may be partly attributed to the inclusion of patients who underwent surgery at the earliest stage of the pandemic. It is likely that some of these patients were referred from previous medical appointments before the restrictions in health services occurred. Thus, it is possible that the real impact of the pandemic on diagnostic delay will be evident only after a long period of time.

The results of the present study showed no differences between the groups in surgical outcomes of patients treated with curative intent. Notably, the number of harvested lymph nodes was higher in the COVID group than in the control group (47.4 *versus* 40.8%; *p*=0.026). However, we found no differences in the extent of lymphadenectomy (D1 *versus* D2), surgical access, or even in the attending surgeons between the two time periods. In contrast, the number of retrieved lymph nodes was much higher in both groups than that recommended by current guidelines (a minimum of 25 by JGCA and 16 by AJCC) (18,19), which is a standard maintained at the institution to ensure the correct assessment of nodal status (13,23).

A higher POC rate is one of the main concerns associated with surgical procedures in patients with cancer during the pandemic (24,25), particularly considering that we expect to receive patients with unfavorable clinical conditions, as mentioned above (26). However, we did not observe an increase in morbidity and mortality in the pandemic cohort compared with the cohort from the previous period. Similar to our results, a previous study evaluated surgical treatments for GC during the COVID-19 pandemic and found no differences in morbidity and mortality rates between the pandemic period and the previous years (27). Moreover, another study including 177 patients with gastrointestinal cancer demonstrated no influence of the pandemic on surgical complications and mortality in the postoperative setting (28).

In our cohort of patients who underwent surgery during the pandemic, only one patient tested positive for COVID-19 in the postoperative period and remained asymptomatic after the diagnosis. However, a study conducted in Brazil demonstrated different results. The study included 99 patients who underwent colorectal surgery, and in the postoperative period, 5 patients tested positive for COVID-19 and exhibited respiratory symptoms. Furthermore, three patients died of complications related to COVID-19 attributed to the severity of the disease and the patient profile (patients with advanced stage, chronic diseases, and low performance status) (5).

This study has some limitations. First, some aspects of the impact of COVID-19 on surgical treatment will only become evident after a longer period of time. Thus, this analysis (which was limited to one year) may provide us with an early view of the pandemic consequences. Considering that the time related to the evolution of symptoms in early to advanced GC is longer than 1 year, the effects of decreased hospital access, abandonment of follow-up, and treatment delay will be verified only in the future. To address this issue, follow-up of cases included in this study must be maintained to provide more evidence for the determination of long-term outcomes.

## CONCLUSION

The number of surgical procedures performed during the pandemic period was lower than the mean number of surgeries performed in previous years. Although the frequency of diagnostic procedures during the COVID-19 pandemic was higher than in previous years, the pandemic did not affect the TNM status, morbidity, and mortality associated with GC surgery. Accordingly, GC surgical treatment may be safely performed with acceptable screening protocol compliance during the pandemic.

## AUTHOR CONTRIBUTIONS

Arneiro AJ, Ramos MFKP and Pereira MA were responsible for the study design, data retrieval, statistical analysis, critical analysis and manuscript drafting. Dias AR was responsible for data retrieval and manuscript review. Ribeiro Junior U, Zilberstein B and Nahas SC were responsible for the critical analysis and manuscript review.

## Figures and Tables

**Table 1:** Clinicopathological and surgical characteristics of patients with GC according to the groups.

Variables	Control group n=535 (%)	COVID group n=83 (%)	*p*
Sex			0.753
Female	203 (37.9)	30 (36.1)	
Male	332 (62.1)	53 (63.9)	
Age (years)			0.732
Mean (SD)	62.1 (12.9)	61.7 (10.3)	
Body mass index (kg/m^2^)			0.666
Mean (SD)	23.7 (4.7)	23.9 (4.5)	
Hemoglobin (g/dL)			0.773
Mean (SD)	11.6 (2.3)	11.5 (2.7)	
Albumin (g/dL)			0.985
Mean (SD)	3.8 (0.6)	3.8 (0.7)	
Neutrophil-lymphocyte ratio (NLR)			0.218
Mean (SD)	3.46 (3.97)	4.26 (5.68)	
Charlson-Deyo Comorbidity Index (CCI)			0.854
0	362 (67.7)	57 (68.7)	
≥1	173 (32.3)	26 (31.3)	
ASA (American Society of Anesthesiologists)			0.409
I/II	341 (63.7)	49 (59)	
III/IV	194 (36.3)	34 (41)	
cTNM			0.833
I	119 (22.2)	15 (18.1)	
II	81 (15.1)	12 (14. 5)	
III	163 (30.5)	27 (32.5)	
IV	172 (32.1)	29 (34.9)	
Type of Surgery			0.005
Curative intent	360 (67.3)	46 (55.4)	
Diagnostic	44 (8.2)	16 (19.3)	
Palliative	131 (24.5)	21 (25.3)	
Lauren's type			0.001
Intestinal	233 (43.6)	25 (30.1)	
Diffuse/Mixed	228 (42.6)	37 (44.6)	
Not specified	74 (13.8)	21 (25.3)	
Preoperative chemotherapy			<0.001
No	435 (81.3)	50 (60.2)	
Yes	100 (18.7)	33 (39.8)	

SD, standard deviation.

**Table 2:** Clinicopathological characteristics of patients with curative GC according to the groups.

Variables	Control group n=358 (%)	COVID group n=46 (%)	*p*
Sex			0.672
Female	144 (40.2)	20 (43.5)	
Male	214 (59.8)	26 (56.5)	
Age (years)			0.954
Mean (SD)	62.4 (13)	62.3 (9.3)	
Body mass index (kg/m^2^)			0.687
Mean (SD)	24.5 (4.5)	24.2 (4.2)	
Hemoglobin (g/dL)			0.811
Mean (SD)	12.1 (2.1)	12.0 (2.6)	
Albumin (g/dL)			0.622
Mean (SD)	3.9 (0.5)	4.0 (0.6)	
Neutrophil-lymphocyte ratio (NLR)			0.135
Mean (SD)	2.82 (2.83)	4.37 (6.83)	
Charlson-Deyo Comorbidity Index (CCI)			0.154
0	233 (65.1)	25 (54.3)	
≥1	125 (34.9)	21 (45.7)	
ASA (American Society of Anesthesiologists)			
I/II	144 (68.2)	28 (60.9)	0.321
III/IV	114 (31.8)	18 (39.1)	
Lauren's type			0.692
Intestinal	195 (54.5)	22 (47.8)	
Diffuse/Mixed	152 (42.5)	23 (50)	
Not specified	11 (3.1)	1 (2.2)	
Tumor size (cm)			0.663
Mean (SD)	4.6 (2.9)	4.5 (2.9)	
pT			0.489
pT1/T2	152 (42.5)	22 (47.8)	
PT3/T4	106 (57.5)	24 (52.2)	
Number of dissected LN			0.026
Mean (SD)	40.8 (18.7)	47.4 (21.3)	
pN			0.769
pN0	163 (45.5)	22 (47.8)	
pN+	195 (54.5)	24 (52.2)	
pM			0.574
pM0	352 (98.3)	45 (97.8)	
pM1	6 (1.7)	1 (2.2)	
pTNM			0.320
I/II	206 (57.5)	30 (65.2)	
III/IV	152 (42.5)	16 (34.8)	

SD, standard deviation; LN, lymph node.

**Table 3:** Surgical characteristics and postoperative outcomes of patients with curative GC according to the groups.

Variables	Control group n=358 (%)	COVID group n=46 (%)	*p*
Access			0.835
Conventional	262 (73.2)	33 (71.7)	
Laparoscopic	96 (26.8)	13 (28.3)	
Type of gastrectomy			0.478
Subtotal	214 (59.8)	30 (65.2)	
Total	144 (40. 2)	16 (34.8)	
Lymphadenectomy			0.921
D1	88 (24.6)	11(23.9)	
D2	270 (75.4)	35 (76.1)	
Length of hospital stay (days)			0.552
Mean (SD)	13.8 (12.1)	12.6 (12.6)	
Median (IQR)	10 (7-15)	8 (6-12.5)	
Postoperative complication (Clavien-Dindo)			0.245
Minor	304 (84.9)	36 (78.3)	
Major	54 (15.1)	10 (21.7)	
30-day mortality			0.362
No	349 (97.5)	44 (95.7)	
Yes	9 (2.5)	2 (4.3)	
90-day mortality			1.000
No	333 (93)	43 (93.5)	
Yes	25 (7)	3 (6.5)	

SD, standard deviation; IQR, interquartile range.

**Table 4:** Postoperative complications of curative GC in the COVID group.

Type of complication	Clavien-Dindo grade				Total complications	
	II	III	IV	V	*n*	%
Gastrointestinal	-	2	-	-	2	15.4
Fistula	1	2	-	1	4	30.8
Infection	2	-	-	-	2	15.4
Cardiac	-	-	2	-	2	15.4
Pulmonary	-	-	2	1	3	23.1
Total	3	4	4	2	13	100.0

**Table 5:** Tests and screening protocol follow-up in the COVID group.

Variables	n=83	%
Preoperative COVID test		
No	19	22.9
Yes (-)	63	75.9
Yes (+)	1	1.2
COVID test interval-surgery (days)		
Median (IQR)	4	
Mean (min-max)	5 (1-21)	
Chest CT		
No	48	57.8
Yes	35	42.2
ICU admission		
No	59	71.1
Yes	24	28.9
COVID test during hospitalization		
No	77	92.8
Yes (-)	5	6.0
Yes (+)	1	1.2
COVID test-follow-up		
No	71	85.5
Yes (-)	9	10.8
Yes (+)	3	3.6

IQR, interquartile range; CT, computed tomography; ICU, intensive care unit.
